# Regional grey matter microstructural changes and volume loss according to disease duration in multiple sclerosis patients

**DOI:** 10.1038/s41598-021-96132-x

**Published:** 2021-08-19

**Authors:** Elisabeth Solana, Eloy Martinez-Heras, Victor Montal, Eduard Vilaplana, Elisabet Lopez-Soley, Joaquim Radua, Nuria Sola-Valls, Carmen Montejo, Yolanda Blanco, Irene Pulido-Valdeolivas, Maria Sepúlveda, Magi Andorra, Joan Berenguer, Pablo Villoslada, E. H. Martinez-Lapiscina, Ferran Prados, Albert Saiz, Juan Fortea, Sara Llufriu

**Affiliations:** 1 Center of Neuroimmunology, Laboratory of Advanced Imaging in Neuroimmunological Diseases, Hospital Clinic Barcelona, Institut d’Investigacions Biomèdiques August Pi i Sunyer (IDIBAPS) and Universitat de Barcelona, Barcelona, Spain; 2 Memory Unit, Department of Neurology, Hospital de la Santa Creu i Sant Pau, Biomedical Research Institute Sant Pau, Universitat Autonoma de Barcelona, Barcelona, Spain; 3 Centro de Investigacion Biomedica en Red de Enfermedades Neurodegenerativas (CIBERNED), Barcelona, Spain; 4 Imaging of Mood and Anxiety Related Disorders (IMARD) Group, Institut d’Investigacions Biomèdiques August Pi i Sunyer (IDIBAPS), Mental Health Research Networking Center (CIBERSAM), Barcelona, Spain; 5 Department of Psychosis Studies, Institute of Psychiatry, Psychology and Neuroscience, King’s College London, London, UK; 6 Centre for Psychiatric Research and Education, Department of Clinical Neuroscience, Karolinska Institutet, Solna, Sweden; 7 Neuroradiology Section, Radiology Service of the Image Diagnosis Center of the Hospital Clinic de Barcelona, Barcelona, Spain; 8 E-health Centre, Universitat Oberta de Catalunya, Barcelona, Spain; 9 Centre for Medical Image Computing (CMIC), Department of Medical Physics and Bioengineering, University College London, London, UK; 10 NMR Research Unit, Queen Square MS Centre, Department of Neuroinflammation, UCL Institute of Neurology, University College London, London, UK

**Keywords:** Multiple sclerosis, Image processing

## Abstract

The spatio-temporal characteristics of grey matter (GM) impairment in multiple sclerosis (MS) are poorly understood. We used a new surface-based diffusion MRI processing tool to investigate regional modifications of microstructure, and we quantified volume loss in GM in a cohort of patients with MS classified into three groups according to disease duration. Additionally, we investigated the relationship between GM changes with disease severity. We studied 54 healthy controls and 247 MS patients classified regarding disease duration: MS1 (less than 5 years, n = 67); MS2 (5–15 years, n = 107); and MS3 (more than15 years, n = 73). We compared GM mean diffusivity (MD), fractional anisotropy (FA) and volume between groups, and estimated their clinical associations. Regional modifications in diffusion measures (MD and FA) and volume did not overlap early in the disease, and became widespread in later phases. We found higher MD in MS1 group, mainly in the temporal cortex, and volume reduction in deep GM and left precuneus. Additional MD changes were evident in cingulate and occipital cortices in the MS2 group, coupled to volume reductions in deep GM and parietal and frontal poles. Changes in MD and volume extended to more than 80% of regions in MS3 group. Conversely, increments in FA, with very low effect size, were observed in the parietal cortex and thalamus in MS1 and MS2 groups, and extended to the frontal lobe in the later group. MD and GM changes were associated with white matter lesion load and with physical and cognitive disability. Microstructural integrity loss and atrophy present differential spatial predominance early in MS and accrual over time, probably due to distinct pathogenic mechanisms that underlie tissue damage.

## Introduction

Multiple sclerosis (MS) is a chronic inflammatory, demyelinating and neurodegenerative disease of the central nervous system that leads to physical and cognitive disability^1^. Early studies considered white matter (WM) damage as a hallmark of MS, although advances in immunopathology and magnetic resonance imaging (MRI) techniques have highlighted the important role of grey matter (GM) damage in the pathogenesis of this disease^2, 3^. Perivenous and confluent demyelinated lesions, as well as subpial cortical demyelination are evident in the GM of MS patients, with diffuse changes only partially related to focal injury^4^. Moreover, neuroaxonal loss in GM may be also related to WM tract degeneration, indicating Wallerian degeneration^5^. Damage to the cortical and deep GM seems to be clinically relevant as it has been linked to the progression of physical and cognitive disability^3, 6, 7^.

Alterations of the GM in MS have been investigated in vivo through different approaches^8–10^. In the context of MS, diffusion tensor imaging (DTI) has the potential to provide quantitative measurements of microstructural changes in GM. However, inconsistencies in the presence and direction (increases or decreases) of the changes in mean diffusivity (MD) and in fractional anisotropy (FA)^7, 10^, highlight the technical challenge present when studying diffusion metrics in the cortex and the complex effect of tissue changes on those metrics^11^. Volumetric studies have also identified patterns of GM atrophy across the brain that appear to predominate in eloquent areas in the early stages of the disease, such as in the thalamus, posterior cingulate cortex and precuneus^8^, and volume loss seems to evolve at different rates over the course of the disease^12, 13^. Atrophy of the cortical GM was predominantly determined by neuronal shrinkage and axonal degeneration. DTI and volume metrics may be sensitive to different pathological processes, and it is not clear whether the rates of microstructural and volumetric changes in GM emerge in parallel or if they follow different trajectories, with differential regional predominance.

It is possible that a new surface-based diffusion methodology may improve the assessment of changes in integrity to the cortical microstructure of the GM in MS. This approach, which reduces the partial volume effects^14–16^, has been applied to other neurodegenerative diseases like Alzheimer's disease and frontotemporal dementia where it offers superior sensitivity in mapping cortical GM changes. Thus, by adopting this methodology, the present study aimed to characterise the regional GM damage, both in cortical and deep GM, analysing the spatial distribution and overlap of diffusion metrics and volume changes in MS patients segregated according to three different periods of the disease course. Moreover, these modifications were mapped to disease severity.

## Results

In this study, 247 MS patients were enrolled (172 women), with a mean age of 42.5 ± 10 years, with 11.6 ± 9.1 years of disease duration and with a median EDSS of 2.0 (range 0–7.0), as well as 54 healthy controls (HC). Demographic and clinical data of the final cohort are summarised in Table 1. The MS patients were divided into three groups according to disease duration, with 67 patients classified as MS1 (less than 5 years of onset), 107 as MS2 (between 5 and 15 years) and 73 patients as MS3 (more than 15 years of disease duration). As expected, the MS3 patients were older than those in the other groups, they suffered greater disability and there was a higher proportion of secondary progressive patients in this group.

**Table 1:** Demographic, clinical, and cognitive data of the study population.

	Healthy controls (n = 54)	Multiple sclerosis patients			
		MS1 (n = 67)	MS2 (n = 107)	MS 3 (n = 73)	*p* value
**Demographic data**					
Female, n (%)	35 (65)	49 (73)	75 (70)	48 (66)	0.709ª
Age (years)	38.5 (10.3)	36.8 (8.9)	40.9 (8.8)	50.2 (8.0)	< 0.001^b^
**Clinical data**					
MS type, n (%)					
Relapsing–remitting	–	67 (100)	102 (95)	58 (79)	< 0.001ª
Secondary progressive	–	0 (0)	5 (5)	15 (21)	
Disease duration, median (IQR)	–	1.1 (0.2–2.9)	10.2 (7.4–12.3)	21.2 (17.2–27.0)	< 0.001^c^
Number of relapses, median (IQR)	–	2 (1–2)	3 (3–5)	5 (3–9)	< 0.001^c^
EDSS score, median (range)	–	1.5 (0–5.5)	1.5 (0–6.5)	2.5 (1.0–7.0)	< 0.001ª
Current use of DMT, n (%)	–	12 (18)	69 (67)	39 (58)	< 0.001ª
Lesion volume (cm^3^), median (IQR)	–	2.73 (1.67–5.13)	4.58 (1.96–8.66)	7.84 (3.88–15.14)	< 0.001^c^
zAttention-Processing speed, median (IQR)	–	− 0.092 (− 0.88–0.5)	0.257 (− 0.33–0.57)	0.087 (− 0.75–0.68)	0.117^c^

Continuous variables are given as the mean (SD). DMT, Disease Modifying Treatment; EDSS, Expanded Disfigability Status Scale; IQR, Interquartile range; MS, multiple sclerosis.

ªChi-squared test.

^b^One-way Anova test; ^c^ Kruskal–Wallis test.

### Regional distribution of GM diffusion and volume changes relative to disease duration

**Figure 1:**
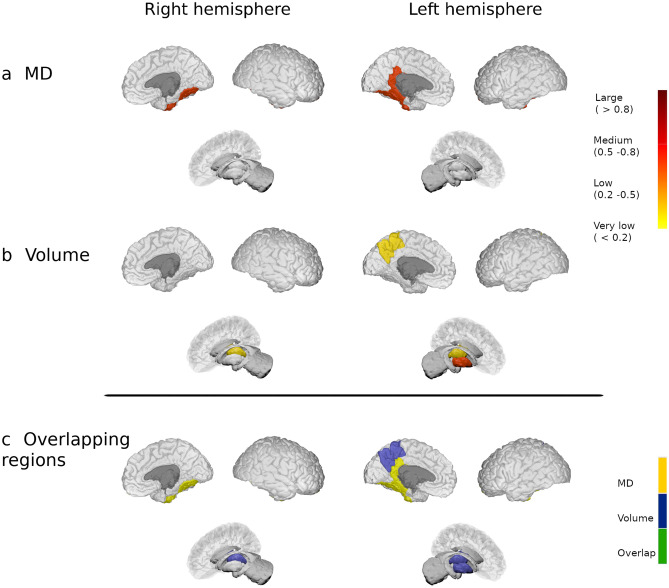
Modifications in patients with less than 5 years of disease duration. Differences in the grey matter mean diffusivity (**a**) and in volume (**b**) between patients from the MS1 group and HC. The colour gradient from yellow to red indicates the effect size ranges. The bottom figure shows the changes in MD (in yellow), volume (in blue) and their overlap (in green). The figure was generated using BrainPainter software^50^.

When compared to HC, there were changes in the MD of the MS1 group over four GM regions (6.3%), mainly involving the temporal lobe, while FA was increased in the lateral occipital and supramarginal cortex from the left hemisphere. GM volume was reduced in deep GM regions and left precuneus. Both MD and GM changes had low-to-moderate effect size, while FA had a very low effect size (corrected *p* < 0.05: Fig. 1 displays MD and volume changes, and Supplementary Fig. 1 shows FA and volume changes for this group of patients).

**Figure 2:**
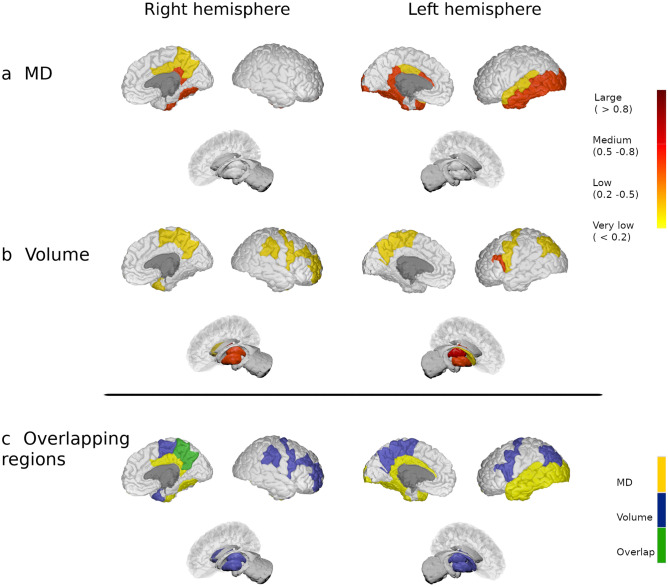
Modifications in patients with 5 to 15 years of disease duration. Differences in the grey matter mean diffusivity (**a**) and in volume (**b**) between MS patients from the MS2 group and HC. The colour gradient from yellow to red indicates the effect size ranges. The bottom figure shows the changes in MD (in yellow), volume (in blue) and their overlap (in green). The figure was generated using BrainPainter software^50^.

In the MS2 group, MD increased in 14 regions (18.4%) relative to the HC, in which most of the regions showed a medium effect size. The largest differences between these groups were evident in areas like the bilateral cingulate, left insula and left parietal cortex (corrected *p* < 0.05). Meanwhile, FA showed low effect size differences between groups, with increased FA in the bilateral putamen and left parietal cortex (corrected *p* < 0.05). In addition, in this group of patients, there was a volume decrease in 19 regions (25%) mainly involving bilateral nuclei of the deep GM and left parietal cortex (corrected *p* < 0.05: Fig. 2 and Supplementary Fig. 2).

**Figure 3:**
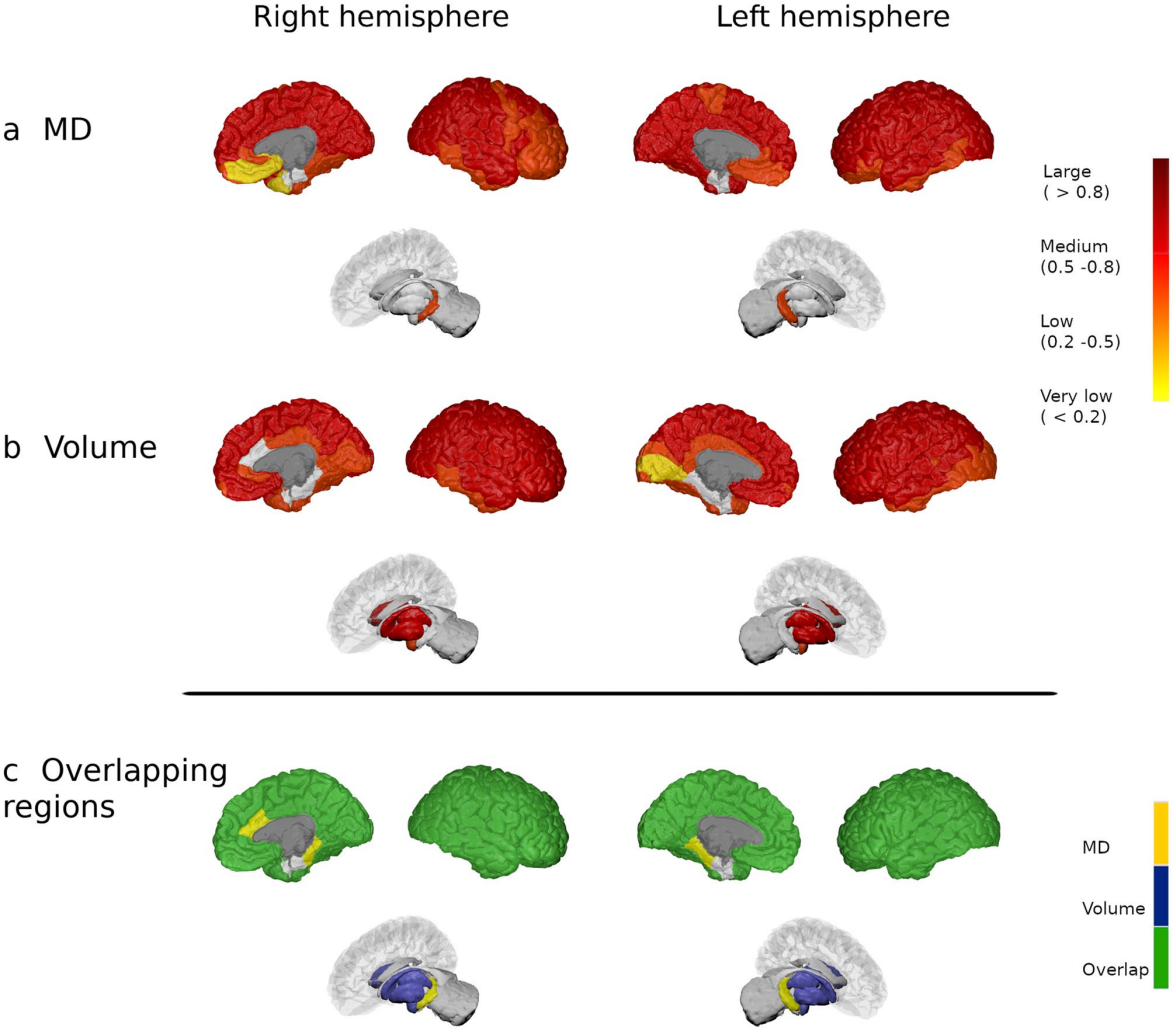
Modifications in patients with more than 15 years of disease duration. Differences in the grey matter mean diffusivity (**a**) and in volume (**b**) between MS patients from the MS3 group and HC. The colour gradient from yellow to red indicates the effect size ranges. The bottom figure shows the changes in MD (in yellow), volume (in blue) and their overlap (in green). The figure was generated using BrainPainter software^50^.

And finally, in the MS3 group, there were 64 regions (84.2%) that showed widespread medium-to-large increase in MD relative to the HC, mainly in the cortex (corrected *p* < 0.05), while MD in the right pallidum was dampened (corrected *p* = 0.001). FA increased in 21 regions (27.6%) involving areas from the bilateral frontal, parietal cortex, and deep GM compared to HC, whereas FA was reduced in the bilateral insula and hippocampus, and also in the left isthmus of the cingulum (corrected *p* < 0.05). Almost all differences had a medium effect-size. Additionally, patients of the MS3 group showed a diffuse reduction in GM volume in 69 regions (90.8%). Bilateral areas involving the insula, frontal and parietal, and right occipital regions displayed the strongest differences between groups (corrected *p* < 0.05: Fig. 3 and Supplementary Fig. 3). Exploring the differences between the older subgroup of HC (47.1 ± 5.7 years, n = 28 subjects) and MS3 (50.2 ± 8.0 years, *p* = 0.063), we found that patients with MS had a higher MD in 63 GM regions (82.9%), higher FA in nine (11.8%) and reduced FA in four (5.3%). Also, patients had widespread reduced volume in 62 nodes (81.6%) throughout the cerebral cortex (corrected *p* < 0.05), similar to the results with the whole HC cohort. Additionally, when we excluded patients with a secondary progressive (SP) MS from the analysis, the results only showed minor modifications. Thus, the frontal cortex showed slightly fewer GM volume reduction in MS2 group and fewer MD changes in the frontal cortex in MS3 group, while the FA in the posterior cingulate was not different in MS3 group compared to HC (corrected *p* < 0.05).

Supplementary Fig. 4 displays areas with significant associations between disease duration and DTI or volume metrics in the whole MS cohort.

### Overlap between diffusion and volume changes relative to disease duration

There was no overlap in regions with increased MD or FA and volume reduction in patients with MS in the first five years of disease duration (Fig. 1 and Supplementary Fig. 1, bottom images). In MS2 patients, only the left transverse temporal and right precuneus showed concomitant MD and volume changes, and bilateral putamen displayed modification in FA and volume (Fig. 2 and Supplementary Fig. 2, bottom images). On the contrary, 55 regions (72.4%) showed both increased MD and reduced volume in patients of the MS3 group, 18 areas (23.7%) increased FA and reduced volume and four regions (5.3%) with reduced FA also showed volume reduction (Fig. 3 and Supplementary Fig. 3, bottom images).

### Association between modifications to DTI and GM atrophy measures with lesion volume and relapses

The increase in MD in MS patients was widely associated with higher WM lesion volume (80.3% of regions, median β = 0.3, 95% confidence interval, CI: 0.19, 0.41: corrected *p* < 0.05). When we measured separately the association between MD and WM lesion volume in each group of patients, both the percentage of regions with significant correlations and the magnitude of the slope decreased in the groups with longer disease duration (Table 2). Increased FA correlated with higher lesion volume in very few regions (5.3% of regions, median β = 0.182, 95% CI: 0.06, 0.3: corrected *p* < 0.05). Whereas, reduced FA in both hippocampus and insula was associated with higher lesion volume (6.6%, median β = − 0.221, 95% CI: − 0.35, − 0.09: corrected *p* < 0.05).

**Table 2:** Association between DTI and GM atrophy measures and white matter lesion volume in each group of MS patients.

GM MRI metrics	Number of regions	Median β (95% CI)	Median *p* value
**Mean diffusivity**			
MS1	4/4	0.485 (0.27, 0.7)	< 0.001
MS2	14/14	0.374 (0.19, 0.56)	< 0.001
MS3	27/64	0.308 (0.11, 0.51)	0.014
**Increased fractional anisotropy**			
MS1	0/2	NS	NS
MS2	0/4	NS	NS
MS3	3/21	0.394 (0.21, 0.58)	0.003
**Decreased fractional anisotropy**			
MS1	0	–	–
MS2	0	–	–
MS3	0/3	NS	NS
**Volume**			
MS1	4/4	− 0.369 (− 0.57, − 0.17)	0.004
MS2	11/19	− 0.25 (− 0.41, − 0.09)	0.021
MS3	12/69	− 0.338 (− 0.54, − 0.14)	0.016

Analyses were performed with the GM regions showing abnormal diffusion or volume in each group of patients.

CI, confidence interval; MS, multiple sclerosis; NS, not significant.

The GM volume reductions were broadly associated with higher WM lesion volume (68.4% of regions, median β = − 0.246, 95% CI: − 0.35, − 0.14: corrected *p* < 0.05). While in the MS1 group, most atrophic regions correlated with WM lesion volume, in the MS2 and MS3 groups the percentage of atrophic areas correlating with lesion volume decreased (Table 2).

### Association between modifications to DTI and GM atrophy measures with clinical characteristics

The increase in MD evident in MS patients was associated with a worse EDSS score (3.9%, median β = 0.229, 95% CI: 0.1, 0.36: corrected *p* < 0.05), but not with the number of relapses. The bilateral hippocampus, lingual and lateral occipital were the areas with the strongest associations. In addition, the MD changes in patients were associated with a worse zAttention-Processing speed in 67.1% of the regions (median β = − 0.191, 95% CI: − 0.32, − 0.06: corrected *p* < 0.05), particularly involving occipital areas and the hippocampus.

FA changes did not correlate with the number of relapses, EDSS or cognitive performance.

The GM volume reductions were associated with more relapses (2.6%, median β = − 0.22, 95% CI: − 0.34, − 0.11: corrected *p* < 0.05) and a higher EDSS score (18.4%, median β = − 0.175, 95% CI: − 0.29, − 0.06: corrected *p* < 0.05) in MS patients. Areas of the deep GM and superior parietal regions were those with the strongest associations. Finally, the GM volume changes were correlated with lower scores of zAttention-Processing speed in 59.2% of the regions (median β = 0.164, 95% CI: 0.05, 0.28: corrected *p* < 0.05), mainly in the bilateral thalamus, posterior cingulate, precentral, left putamen, and right superior parietal cortex.

## Discussion

By applying a new diffusion MRI processing pipeline to GM regions in combination with volumetric measurements, this study reports anatomical changes to cortical and deep brain structures that are related to disease duration in patients with MS. We found regional modifications of microstructural tissue architecture and tissue loss within the first five years of the illness. Early in the disease, changes to microstructural barriers predominated in the cortex, while volume loss was mainly linked to deep brain structures and precuneus, without overlap between these changes. GM modifications became more evident and widespread with longer disease duration. Thus, more than fifteen years after disease onset, almost all the cortex and deep GM displayed extensive damage, which was associated with physical and cognitive disability.

Our results indicate that MD, rather than FA, is sensitive to GM damage in patients with MS already at the beginning of the illness. The higher MD values in patients during the first five years of the disease were mainly limited to the temporal and cingulate cortices. With longer disease duration, MD also increased in other areas of the temporal and occipital cortex, and later on, its rise was seen in almost the entire brain with relative sparing of the deep GM structures. On the contrary, modifications in FA in patients with shorter disease durations had very low effect size, they were scarce and mainly related to FA increments. This is probably because in the cortex, the relative damage to the perpendicular or parallel axons can influence the directionality of FA modifications, and although demyelinated cortex could present higher FA values compared to normal-appearing cortex, the opposite effect of injury to both types of axons can underestimate the differences with healthy cortex^11^. Also, the FA index is particularly influenced by the presence of crossing fibres or other crossing geometries^17, 18^. MD is insensitive to the orientation of the tissue at the voxel length scale, a fact that has promoted its use in the study of GM damage in several neurologic diseases^19^. The early diffusion damage to the cerebral cortex supports the susceptibility of these areas to the loss of integrity of microstructural barriers^20^ facilitating free diffusivity^21^. Post-mortem studies have related MD values in the WM with myelin content, and with axonal count to a lesser extent^22^ but results in the cortex have not shown a straightforward relationship with histology, probably in part due to the complex structure of the cortex and the lower amount of myelin^23^. The modifications observed here may be linked to the early cortical damage, at least in part, driven by meningeal inflammation^24^, although other pathological factors may play a role^7, 10^ including retrograde Wallerian degeneration from WM damage as pointed out by the association between WM lesion load and DTI changes.

Brain atrophy is one of the main features of MS disease progression, and it is thought to be uneven across the brain, indicating that some regions are more vulnerable to damage than others^8, 9, 12^. We found that the bilateral thalamus, right putamen and left precuneus were the first regions to lose volume, after which, atrophy accrued in other deep GM nuclei, inferior parietal and frontal pole. After considerable years of disease duration, almost the entire brain showed GM atrophy in patients with MS. These results confirm previous studies reporting the precuneus and thalamus as the first regions to become atrophic, reflecting the susceptibility of these highly-connected regions to MS damage^25–27^. Later on, other deep structures and multimodal areas become affected. The vulnerability of these regions to neuroaxonal loss and neuronal shrinkage could be explained by their widespread connectivity with other brain areas, occurring both through marked anterograde^28^ and Wallerian or retrograde degeneration^29^, that may be specially relevant in early stages as shown by the association we found between atrophy and WM lesion volume. Moreover, other pathological events including the presence of both diffuse oxidative injury and neurodegenerative mechanisms such as iron accumulation^25, 26^ can promote GM volume loss.

One relevant finding from the analysis carried out here is the lack of overlap between the areas with changes in diffusion MRI and volume in the first years of the disease course, with a cortical preference for MD changes and the deep GM for volume modifications. Moreover, MD effect sizes were stronger than those of volume loss at the beginning (in MS1 group), suggesting that MD is initially more sensitive to cortical damage. In the later phases of MS, cortical volume reductions also emerged, as well as increased FA in deep GM, frontal and parietal lobes. Both DTI and volume modifications were associated with the WM lesion load, but not significantly with relapses. These results and the lack of regional co-localization endorses the hypothesis that neurodegeneration in the cortex is partially independent of cortical demyelination^30^. However, future work is necessary to endorse this hypothesis.

Damage in cortical and deep GM has been associated with the presence of clinical and cognitive disability^3, 6, 7, 13^. Indeed, in this study, both increased MD and reduced GM volume in specific areas were correlated with worse physical and, specially, with cognitive performance. In general, increased MD in occipital and bilateral hippocampus and volume reduction in thalamus and other deep GM, superior parietal and posterior cingulate cortex were the regions most strongly associated with worse attention, working memory and psychomotor speed, supporting the relevance of these multimodal areas and their connections to maintain superior functions^31^.

This study has several strengths. First, we evaluated the cortical damage using an improved technique to compute diffusion metrics that has proven to be sensitive to describe cortical changes in other neurological diseases. Second, we combined two approaches to characterise the damage to the GM, complementary methods that provide information on different pathological changes in these areas. The results support the use of these techniques in clinical trials to assess neuroprotective therapies. Our study also has some limitations. We set out to understand the GM involvement in MS in terms of disease duration, but we did not obtain differential information on lesions and normal-appearing GM. Therefore, future studies should evaluate the differential impact of focal cortical demyelination and cortical microarchitecture changes. In this sense, the use of advanced multicompartment diffusion MRI models, such as Neurite orientation dispersion and density imaging (NODDI), may provide larger sensitivity to disentangle pathological from the normal GM tissue and to minimise the effects of partial volume effects of cerebrospinal fluid signal across cortical microarchitecture^32, 33^. Although the patients with a longer disease duration were older than the HC, the results remained unchanged when we balanced the cohorts for age by comparing MRI metrics with the older HC individuals. We are aware that our cohort of MS patients was mainly composed of relapsing–remitting patients, and therefore limits the generalizability of the results to more progressive phenotypes. Nevertheless, this phenotype is the most common one, thanks to the lower rates of worsening and progression to SP phases in the treatment era^34^, and the results without the group of progressive patients were maintained. Attention and processing speed were assessed with the SDMT and PASAT, thus the use of a full neuropsychological battery will be necessary to support our findings on other cognitive domains. Finally, we followed a cross-sectional approach that allows patients with a wide range of disease duration to be compared, although further studies using longitudinal data will be needed to shed light on brain MS damage dynamics in a prospective manner.

To conclude, patients with MS suffer local modifications to their tissue microstructure and a loss of GM volume early in the disease course, which become widespread at later stages and that are related to disability. Initially, changes to diffusion metrics predominate in the cortex while atrophy is more restricted to deep GM regions, with weak spatial co-localization. Such results provide relevant information on the spatial modifications of tissue changes and support the presence of several pathogenic mechanisms underlying the evolution of damage over time, accounting for GM impairment in MS, although longitudinal studies are needed to further understand the dynamics of those changes.

## Methods

### Participants

For this cross-sectional study, we included a cohort of 271 patients with relapsing–remitting or SPMS according to the 2010 McDonald criteria^35^ prospectively recruited at the MS Unit in the Hospital Clinic of Barcelona, as well as 54 HC without any prior or present history of neurological or psychiatric conditions. Patients were aged 18 to 65 years old, did not present any relapse or received corticosteroids in the last 30 days, and had no modifications in the disease modifying therapy in the last three months. To better describe GM changes during the disease, we classified patients into three groups according to their disease duration: less than 5 years from disease onset (MS1); from 5 to 15 years (MS2); and more than 15 years of disease duration (MS3)^36^.

Physical disability was evaluated in all patients using the Expanded Disability Status Scale (EDSS)^35^, and in 214 of them, attention and information processing speed was assessed using the Symbol Digit Modalities Test and Paced Auditory Serial Addition Test from the Brief Repeatable Battery of Neuropsychological tests^37^. We calculated a mean z-score of these two tests (zAttention-Processing speed) using the age-and education-adjusted values of a healthy Spanish cohort^38^.

The Ethics Committee of the Hospital Clinic of Barcelona approved the study and all the participants signed an informed consent before enrolment into the study. All procedures were performed according to the principles of the Helsinki Declaration.

### MRI acquisition and processing

#### Structural and diffusion MRI acquisition

MRI was performed on a 3 T Magnetom Trio (SIEMENS, Erlangen, Germany) scanner with a 32 channel phased-array head coil. The protocol included structural 3D-Magnetization Prepared Rapid Acquisition Gradient Echo (MPRAGE), 3D-T2 fluid-attenuated inversion recovery (FLAIR) and diffusion-weighted imaging (DWI) sequences. DWI was acquired with two different sequence parameters. A detailed description of all sequences used in this study is available in the Supplementary material^12, 39^.

#### Grey matter volume processing

**Figure 4:**
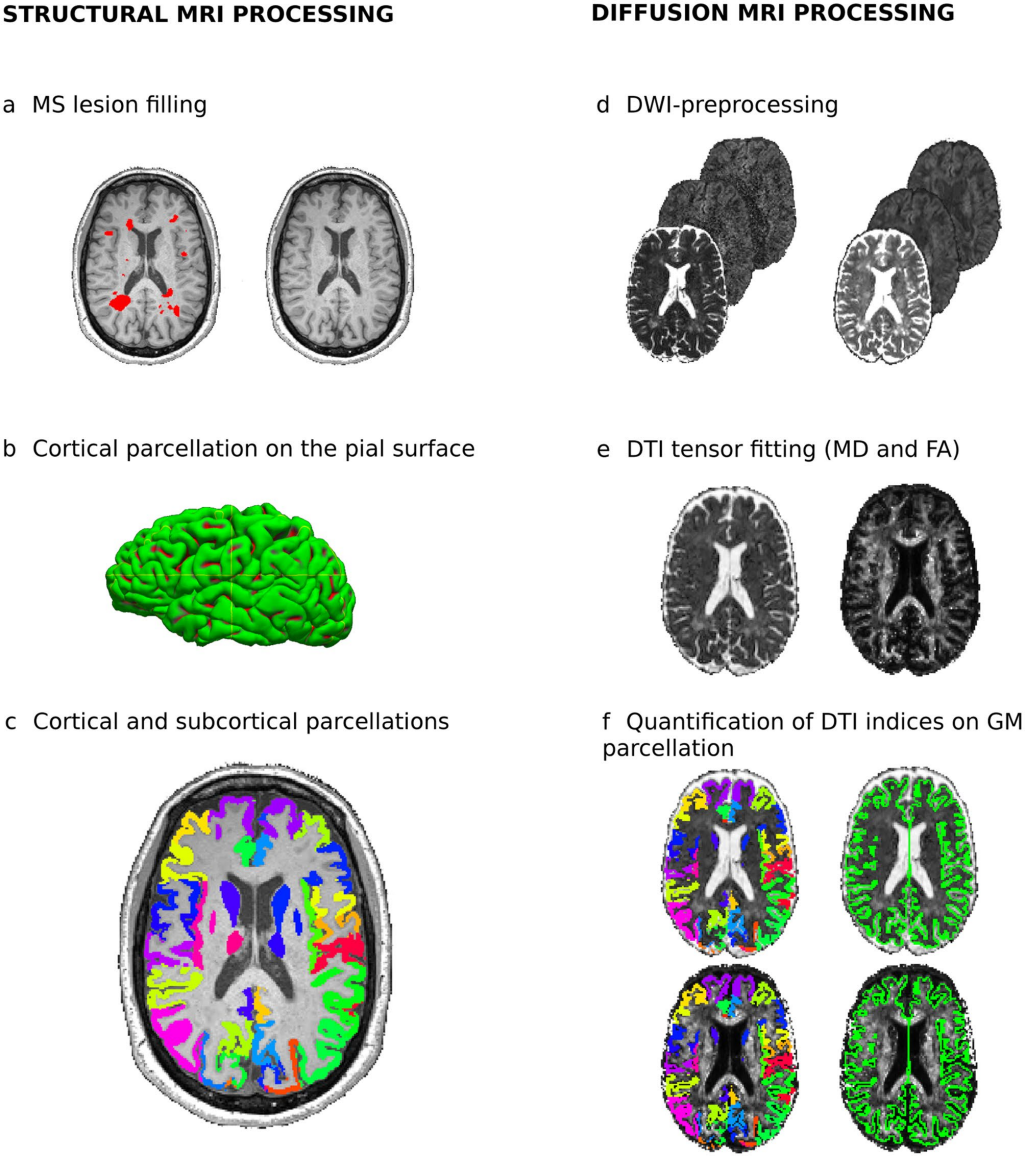
Flowchart for the structural (left) and diffusion MRI processing (right). To quantify GM volume (left), WM lesions in the 3D-MPRAGE were segmented using 3D-FLAIR as a reference and a WM lesion-filling approach was applied (**a**). We parcellated both the cortical pial surface (**b**), and the cortical and subcortical GM regions, to obtain volumetric values in 76 regions. To calculate the diffusion metrics in each region (right), after performing the standard diffusion pre-processing steps (**d**) we obtained the mean diffusivity and fractional anisotropy measures (**e**), which was realigned to the cortical and subcortical surface on the diffusion tensor imaging space (**f**).

Before computing GM volume, WM lesions were segmented in 3D-MPRAGE space with ITK-SNAP Software (http://www.itksnap.org/pmwiki/pmwiki.php). We used a linear registration of the 3D-FLAIR image to align with 3D-MPRAGE to improve both the identification and delineation of lesions. Subsequently, we applied a WM lesion-filling approach^40^ and obtained 76 GM regions using the Mindboggle software (https://mindboggle.info), applying the Desikan-Killiany-Tourville cortical labelling atlas (31 cortical labels per hemisphere)^41^, and the automated subcortical segmentation offered by the FSL-FIRST package for seven subcortical regions in both hemispheres^42^. The resulting cortical surface parcellation was visually checked and fixed to guarantee the quality of the cortical segmentation labels for further statistical analysis. We excluded 24 patients due to segmentation errors in the cortical surface reconstruction. Finally, we normalised the 76 GM volumes using a volumetric scaling factor provided by SIENAX to reduce the effect of the variability in head-size for quantification^43^. The flowchart for GM volumetric processing is summarised in Fig. 4 (left panel).

#### Grey matter diffusion processing

The diffusion MRI pre-processing pipeline was described in detail elsewhere^31, 39^, and it involved the following steps: DWI denoising, Gibbs ringing correction, motion-induced distortion correction, a phase unwrapping procedure to correct for geometric distortion and bias field correction^44^. Subsequently, the diffusion tensor metrics (MD and FA) were obtained^45^.

The GM cortical reconstruction process was performed using in-house surface-based approach developed to measure the microstructural changes in neurodegenerative disorders^14, 15, 46^. We used Freesurfer tools to calculate the midpoint of each individual’s cortical surface in order to avoid contamination by adjacent WM and cerebrospinal fluid^14, 15^. We then registered the T1-weighted image to the pre-processed diffusion data using a boundary-driven algorithm to realign both the cortical surface mask generated and the 76 GM labels on the DTI maps of each subject^47^. Likewise, we selected only those voxels labelled as the midpoint cortical mask to obtain the average of the diffusion metrics for each label. Due to the low-resolution of the DWI voxel, we assume that information from most cortical layers are inherently included in DTI measures. We then eroded each of the 14 deep GM structures using a 3 mm box kernel to minimise the partial volume effects, and computed the MD and FA average within these masks. The GM diffusion processing pipeline is summarised in Fig. 4 (right panel).

Diffusion and volumetric data were harmonised using the ComBat function in R software^48, 49^ to reduce the variability of these measures related to the use of different acquisition protocols.

### Statistical analysis

We described the demographic, clinical and neuroimaging data with the mean and standard deviation (SD, for continuous data), or as absolute numbers and proportions (for categorical data), assessing the former’s normal distribution with histograms and Shapiro–Wilk tests. We analysed the demographic and clinical differences among groups with a Chi-squared or Kruskal–Wallis H test, using Dunn’s test when necessary, and we compared the GM diffusion and volumetric data with one-way ANOVA, using Tukey HSD test for the two-group differences (HC vs. MS1, MS2 and MS3). Furthermore, we described the effect size of these differences using Hedges’ g. To ensure that age differences between the HC and MS3 group did not confound the results, we additionally calculated the changes in the MRI metrics between these two groups with a Student t-test or Mann Whitney U Test, selecting only the older HC subjects. Finally, we used linear regression to analyse the association between GM diffusion or volume and disease duration, WM lesion volume (including a post-hoc analysis for each group of patients), and measures of disease severity (number of relapses, EDSS, and the mean z-Attention-Processing speed score in the whole sample of MS patients). For easier interpretation, all variables were standardised using the mean and SD.

In all the analyses, we included age and gender as covariates to control for its potential influence on the results. We used the false discovery rate (FDR) to correct for multiple comparisons, and we set the significance level at a corrected *p* < 0.05. The R Statistical Software (version 3.6.1, www.R-project.org) was used for all statistical analyses.

## Supplementary Information

Supplementary Information.

## Data Availability

The datasets generated during and/or analysed during the current study are available from the corresponding author on reasonable request.
