# Supplementary material for: Regional grey matter microstructural changes and volume loss according to disease duration in multiple sclerosis patients
Source: Sci Rep. 2021 Aug 19;11:16805. doi: 10.1038/s41598-021-96132-x (PMC8376987; doi:10.1038/s41598-021-96132-x)
Supplement: Supplementary file 1 — Supplementary Information. [file 41598_2021_96132_MOESM1_ESM.pdf]

Regional grey matter microstructural changes and volume loss according to disease duration in multiple sclerosis patients

Elisabeth Solana, Eloy Martinez-Heras, Victor Montal, Eduard Vilaplana, Elisabet Lopez-Soley, Joaquim Radua, Nuria Sola-Valls, Carmen Montejo, Yolanda Blanco, Irene Pulido-Valdeolivas, Maria Sepúlveda, Magi Andorra, Joan Berenguer, Pablo Villoslada, Martinez-Lapiscina EH, Ferran Prados, Albert Saiz, Juan Fortea, Sara Llufriu.

Supplementary material

Acquisition details:

In part of the cohort (n = 207 participants), the 3D-structural image was acquired with TR = 1800 ms; TE = 3.01 ms; TI = 900 ms; 240 sagittal slices with 0.94 mm isotropic voxel size and a 256 × 256 matrix size; and the 3D-T2 FLAIR with TR = 5000 ms; TE = 304 ms; TI = 1800 ms; 192 sagittal slices with 0.94 mm isotropic voxel size and a 256 × 256 matrix size. The DWI had a TR = 14800 ms; TE = 103 ms; 100 contiguous axial slices; 1.5 mm isotropic voxel size; a 154 × 154 matrix size; b value = 1000 s/mm^2; 60 diffusion encoding directions and a single baseline image acquired at 0 s/mm2. The remaining participants (n = 94) had a 3D-structural image with TR = 1970 ms; TE = 2.41 ms; TI = 1050 ms; 208 sagittal slices with 0.9 mm isotropic voxel size and a 256 x 256 matrix size; and the 3D-T2 FLAIR with TR = 5000 ms; TE = 393 ms; TI = 1800 ms; 208 sagittal slices with 0.9 mm isotropic voxel size and a 256 × 256 matrix size. The DWI acquisition protocol was: TR = 12600 ms; TE = 112 ms; 80 contiguous axial slices; 2 mm isotropic voxel size; a 120 × 120 matrix size; b value = 1500 s/mm^2; 70 diffusion encoding directions and a single baseline image acquired at 0 s/mm^2. In addition, field map images were

generated for all participants to correct the distortions caused by field inhomogeneities (TE 1/TE 2 = 4.92/7.38 ms, with the same slice prescription, slice thickness and field of view as the DWI sequence).

Supplementary Figures

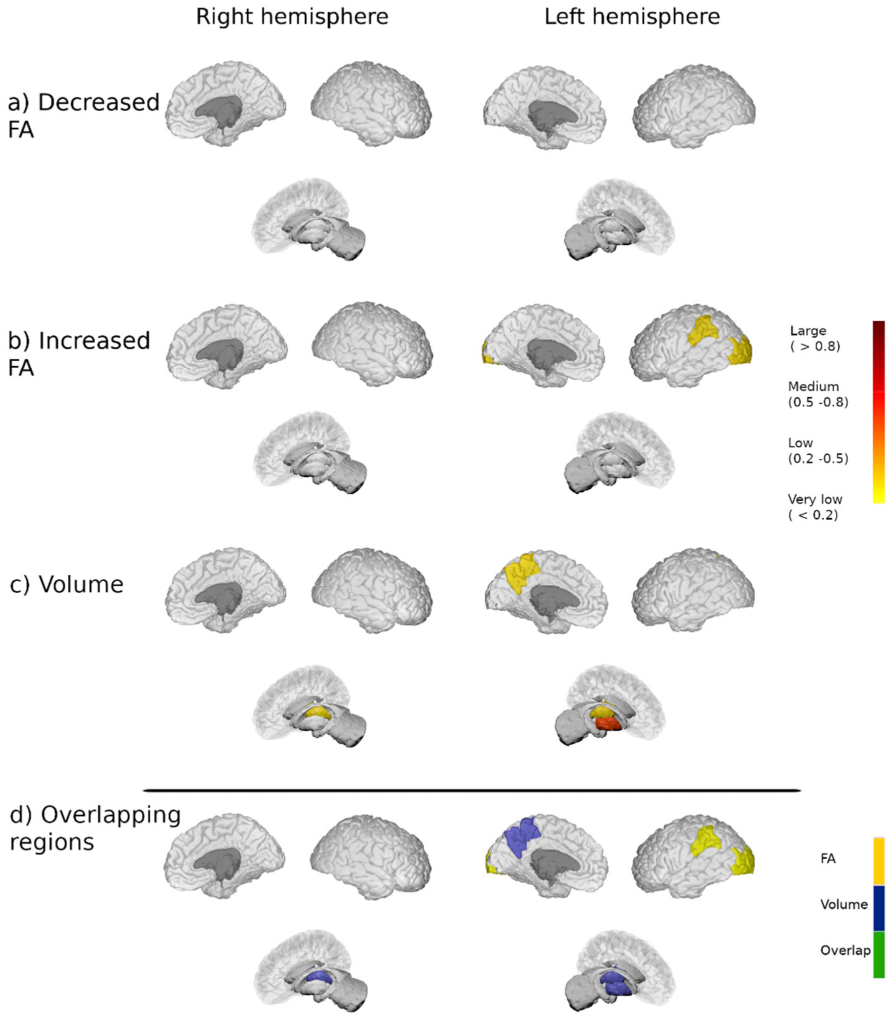

Supplementary Fig. 1. Modifications in patients with less than 5 years of disease duration. Differences in the grey matter by fractional anisotropy measures (a, b) and in volume (c) between patients from the MS1 group and HC. The colour gradient from yellow to red indicates the effect size ranges. The bottom figure shows the changes in FA (in yellow), volume (in blue) and their overlap (in green). The figure was generated using BrainPainter software.[49]

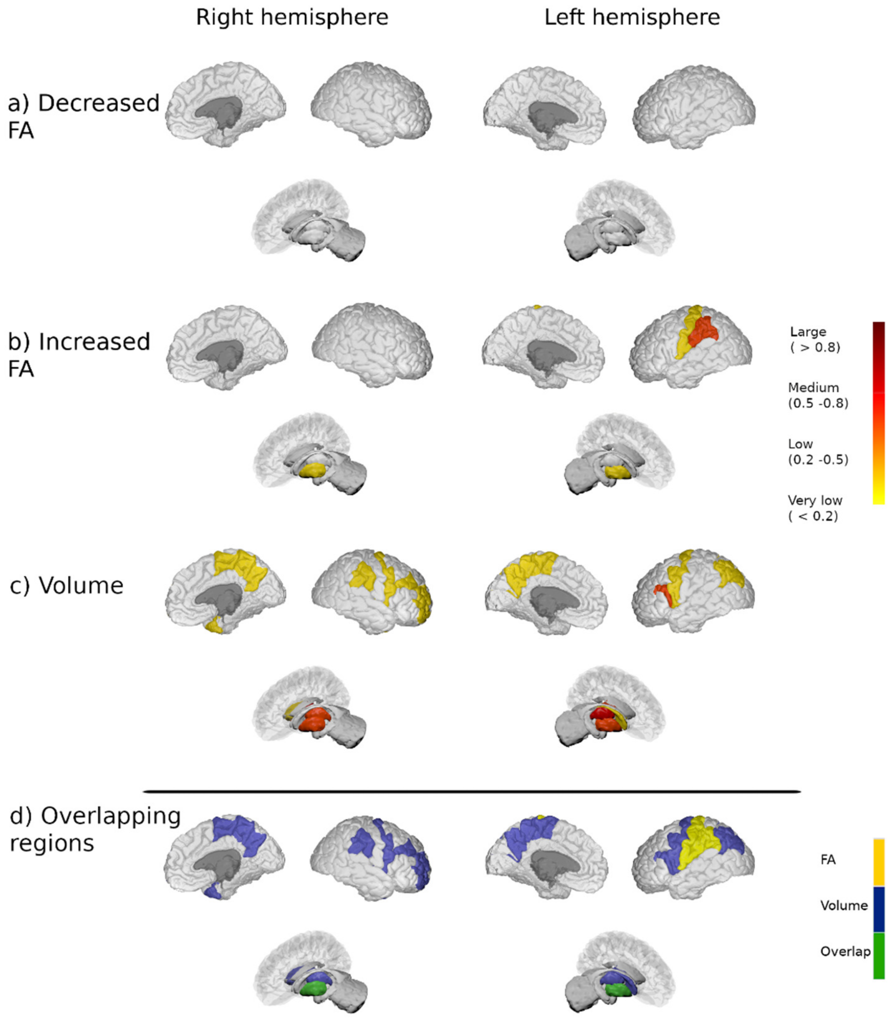

Supplementary Fig. 2. Modifications in patients with 5 to 15 years of disease duration. Differences in the grey matter by fractional anisotropy measures (a, b) and in volume (c) between patients from the MS1 group and HC. The colour gradient from yellow to red indicates the effect size ranges. The bottom figure shows the changes in FA (in yellow), volume (in blue) and their overlap (in green). The figure was generated using BrainPainter software.[49]

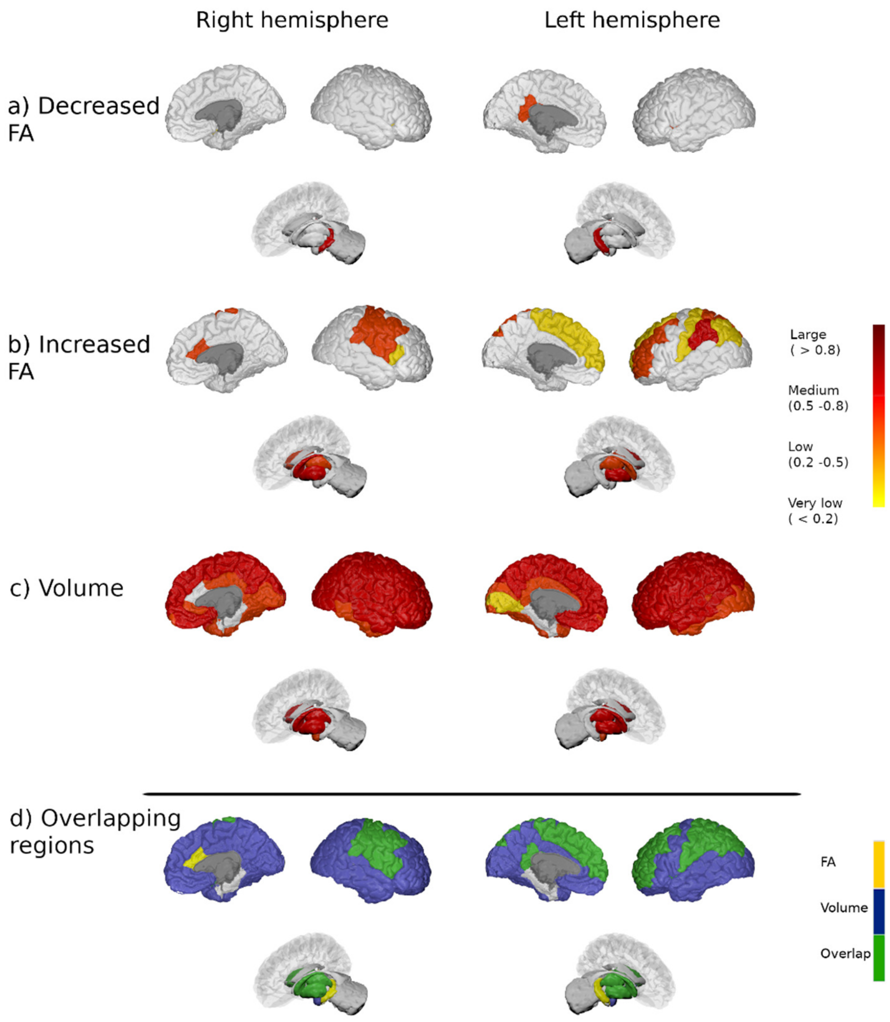

Supplementary Fig. 3. Modifications in patients with more than 15 years of disease duration. Differences in the grey matter by fractional anisotropy measures (a, b) and in volume (b) between MS patients from the MS2 group and HC. The colour gradient from yellow to red indicates the effect size ranges. The bottom figure shows the changes in FA (in yellow), volume (in blue) and their overlap (in green). The figure was generated using BrainPainter software.[49]

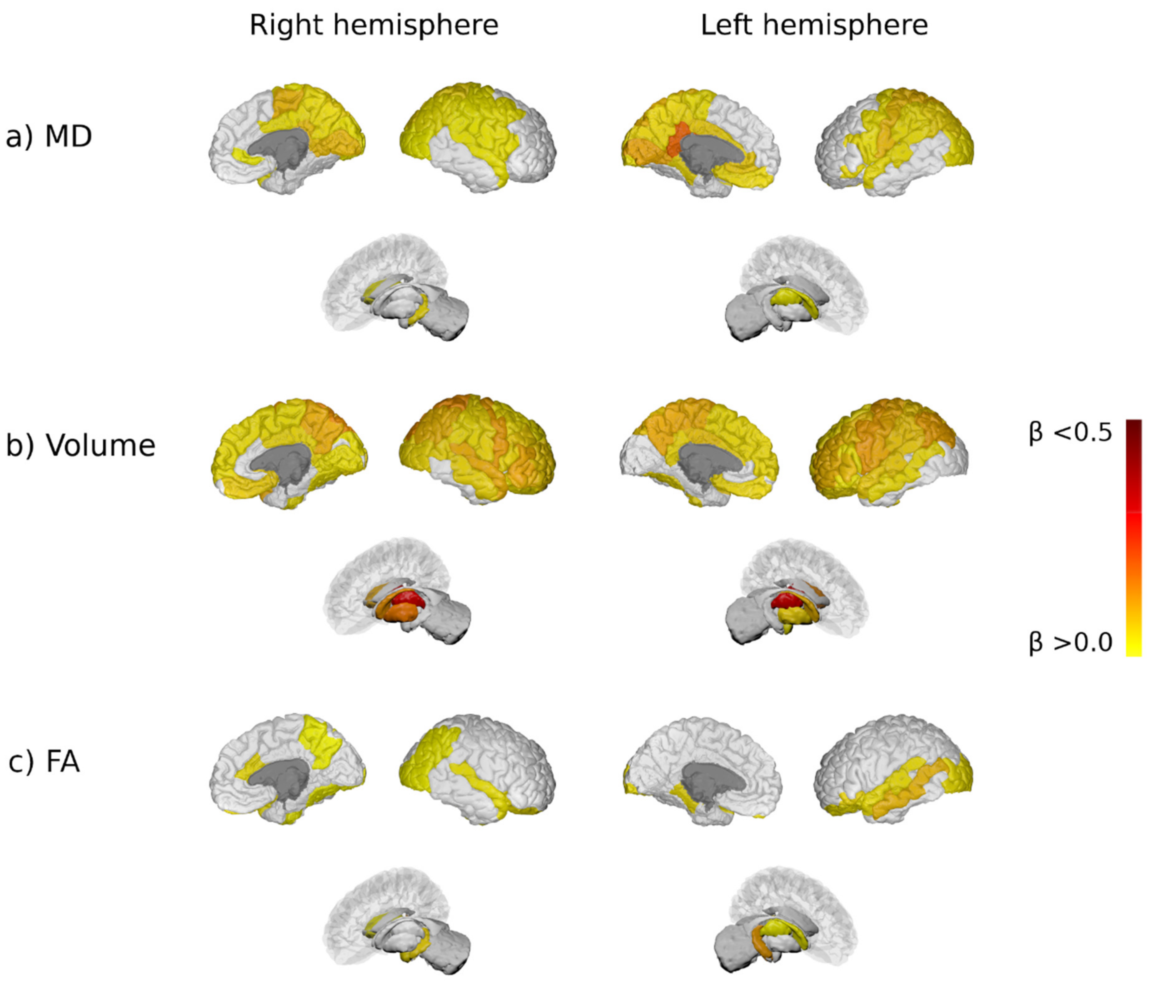

Supplementary Fig. 4. Association between (a) MD values, (b) GM volume and (c) FA indices with disease duration. The colour gradient from yellow to red indicates the slope of the regression line. The figure was generated using BrainPainter software.[49]
